# Two Sides of One Coin: Affective Psychosis vs. Non-Affective Psychosis in Teenagers

**DOI:** 10.3390/children13020251

**Published:** 2026-02-11

**Authors:** Ana Maria Stirbu, Andra Mihaela Lăptoiu, Lucia Emanuela Andrei, Raluca Grozavescu, Florina Rad

**Affiliations:** 1 Child and Adolescent Psychiatry Department, Carol Davila University of Medicine and Pharmacy Bucharest, 050474 Bucharest, Romania; 2 “Professor Doctor Alexandru Obregia” Psychiatric Clinical Hospital, 41915 Bucharest, Romania; ana-maria.stirbu@rez.umfcd.ro

**Keywords:** adolescent psychosis, affective psychosis, non-affective psychosis, treatment, academic performance

## Abstract

Background/Objectives: Psychotic episodes in adolescents may present as affective psychosis (AP) or non-affective psychosis (NAP), which are clinically overlapping. This single-center, retrospective study aimed to describe and compare adolescents with AP and NAP regarding demographic characteristics, family history, academic functioning, and treatment patterns. Methods: We retrospectively analyzed medical records of 171 patients aged 11–18 years admitted to the “Prof. Dr. Alexandru Obregia” Clinical Psychiatric Hospital, Bucharest, between 2020 and 2024. Descriptive statistics and univariate analyses were performed to explore associations between episode type and selected clinical and sociodemographic variables. An exploratory multivariate logistic regression was conducted to assess whether associations observed in univariate analyses remained stable after adjustment for relevant covariates. Treatment-related variables were analyzed descriptively and were not included as predictors in the regression model. Results: AP was more frequent in females (53.2%), whereas NAP predominated in males (62.8%, *p* = 0.036). Trends toward higher rates of psychiatric family history and better academic performance were observed in the AP group, while school dropout was more frequent in NAP, although these differences did not reach statistical significance. Mood stabilizers were prescribed more frequently in adolescents with AP, reflecting clinical practice patterns; however, this association did not reach statistical significance and was interpreted descriptively. In multivariate analysis, gender remained significantly associated with episode type. Conclusions: This retrospective study identifies descriptive differences between adolescent AP and NAP in gender distribution, family psychiatric history, academic functioning, and treatment patterns. Findings are exploratory and do not imply causality. Results highlight areas for further research and underscore the importance of considering clinical subtype and functional outcomes when planning interventions in adolescent psychosis.

## 1. Introduction

Lately, the field of psychiatry has shifted from a strictly categorical classification system to a dimensional approach, with particular attention to psychosis. Recent discussions have characterized psychosis and affective psychosis as distinct but overlapping entities within the same spectrum [1,2], highlighting shared features and complex interrelations among psychotic and affective traits. This dimensional framework encompasses conditions such as depression with psychotic symptoms, mania with psychotic symptoms, and schizoaffective disorder. Due to the limited data specifically addressing affective psychosis, most available evidence derives from studies that include both affective and non-affective forms. For clarity, we designated the group experiencing affective psychosis as “AP” and the group experiencing non-affective psychosis as “NAP.”

Gender differences have been consistently reported in the literature, with females more frequently affected by AP, while NAP shows comparable prevalence across genders [1,3,4]. Cognitive and functional domains have also been highlighted as important correlates of outcome in psychotic disorders. Previous studies have described impairments in intelligence, verbal comprehension, attention, working memory, reasoning, and executive functioning, particularly in individuals with non-affective psychosis, which motivated the inclusion of academic functioning as an exploratory variable in our cohort [5,6].

Several potential risk factors have been discussed in the literature in relation to different psychosis subtypes, particularly for non-affective psychosis, including urbanicity, immigration, perinatal complications, and head injuries [7,8]. Prenatal infections have also been proposed as possible contributors to affective psychosis. However, these factors are not specific to either subgroup and show considerable overlap with other psychiatric conditions [7]. Pharmacological approaches also differ between subtypes, with affective psychosis generally responding more favorably to mood stabilizers, while non-affective psychosis is primarily treated with antipsychotic medication [3,9,10].

Although few studies have focused specifically on adolescents aged 11–18 years, this developmental period is critical for understanding the early course of psychotic disorders and optimizing early interventions, which have been associated with more favorable outcomes [11]. To our knowledge, no single-center study has comprehensively compared affective and non-affective psychosis in this adolescent population.

The aim of this study is to descriptively compare adolescents with affective psychosis (AP) and non-affective psychosis (NAP) in terms of demographic characteristics, personal and familial psychiatric background, academic functioning, and treatment patterns. Using univariate and exploratory multivariate analyses, we examined associations between episode type and these clinically relevant variables. Given the retrospective, single-center design, all analyses are exploratory and intended to describe patterns of association rather than etiological or causal mechanisms.

## 2. Material and Methods

This retrospective single-center study included patients aged 11 to 18 years, hospitalized at the Clinical Psychiatric Hospital ‘Professor Dr. Alexandru Obregia’, Bucharest, over a five-year period (January 2020 to December 2024). Patients were included if they had a confirmed clinical diagnosis of either affective psychosis (AP), characterized by mood-congruent psychotic symptoms such as mania or depression with psychotic features, or non-affective psychosis (NAP), defined as psychotic disorders without prominent mood symptoms, including schizophrenia spectrum disorders. Diagnoses were established by board-certified child and adolescent psychiatrists following DSM-5 criteria. No formal interrater reliability assessment was performed due to the retrospective, chart-based design.

Most participants had detailed medical records; however, due to the retrospective nature and inclusion of patients from foster care, some information may be incomplete or biased. No patients were identified as having transitioned from AP to NAP before the age of 18; however, transitions cannot be fully excluded given the study design. Exclusion criteria included moderate or severe intellectual disability, substance-induced psychosis, secondary psychosis due to organic causes, affective disorders without psychotic features, and insufficient clinical documentation.

Data were collected from medical records and caregiver interviews, including:

- Demographic variables: age, gender, area of residence.
- Clinical variables: type of psychotic episode, initial treatment, use of benzodiazepines and mood stabilizers.
- Medical and family history: personal pathological and physiological history, neuropsychiatric and somatic antecedents, family structure.
- Academic performance: school attendance and performance.

Data analysis was performed using IBM SPSS Statistics (version 21.0; IBM Corp., Armonk, NY, USA) and JASP (version 0.95.1; JASP Team, Amsterdam, The Netherlands). Descriptive statistics were computed for continuous variables (mean, standard deviation, and distribution analysis including skewness) and categorical variables (frequencies and percentages). Age was treated as a continuous variable with distribution assessed via descriptive statistics. Associations between categorical variables were tested using Pearson Chi-square tests. We performed an exploratory multiple regression model to see if the significance of the variables analyzed was preserved. Multicollinearity was assessed by variance inflation factor (<1.5) and tolerance (>0.1). Bootstrapped coefficients were calculated through empirical bootstrapping (available in JASP) from 1000 resamples.

The threshold for statistical significance was set at *p* < 0.05.

To complement the statistical analysis, visual representations were created using pie charts and bar charts to compare the distribution of key variables between the two groups of psychotic episodes.

## 3. Results

### 3.1. Descriptive Analysis

**Figure 1:**
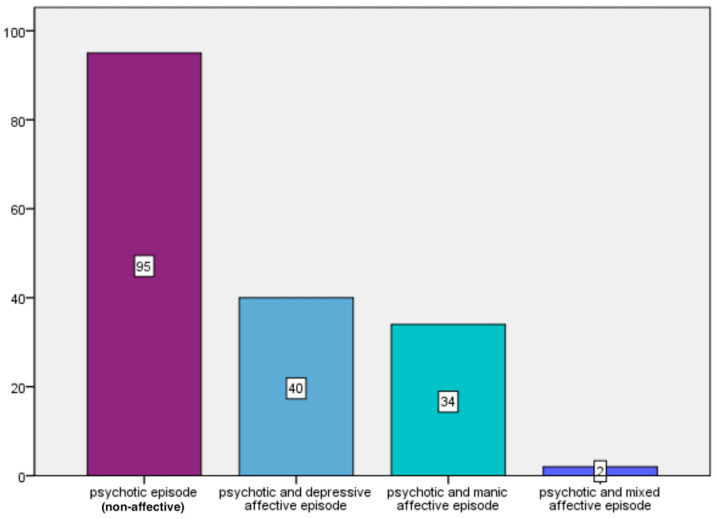
Distribution of patients according to episode type (Total = 171 patients).

A total of 171 participants met the inclusion criteria. Among them, 95 were diagnosed with non-affective psychosis (55.5%) and 76 with affective psychosis (44.5%), including 40 with depressive symptoms, 34 with manic symptoms, and 2 with mixed symptoms (Figure 1).

**Figure 2:**
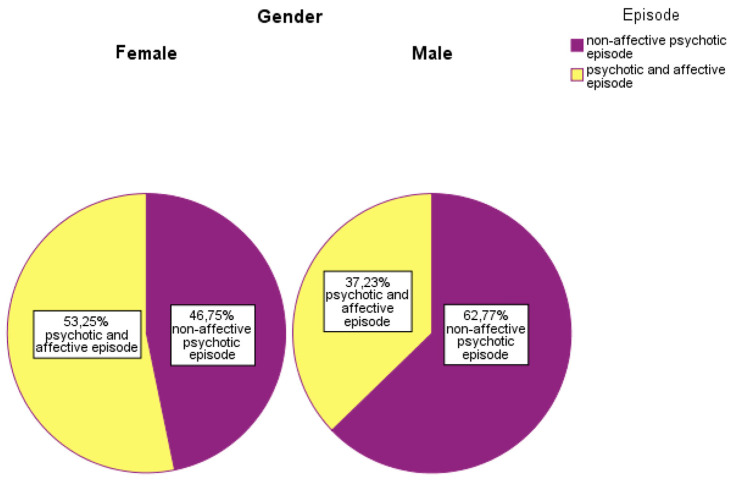
Distribution of patients according to gender and episode type.

Regarding gender, 62.77% of male patients had non-affective psychosis, while 37.23% had affective psychosis. Among female patients, 53.25% had affective psychosis and 46.75% non-affective psychosis (Figure 2).

Age ranged from 11 to 18 years, with a mean of 15.39 ± 1.617 years. The age distribution was slightly skewed towards younger participants, with most aged 14–17 years.

Family history showed that 59.21% of affective psychosis patients had relatives with psychiatric disorders, compared to 50.53% in the non-affective group. The main conditions observed in relatives were anxiety–depressive disorders, psychotic disorders, and chronic alcoholism.

Personal medical history indicated that 23.16% of non-affective psychosis patients had no prior medical conditions, compared to 36.84% in the affective psychosis group. Neurodevelopmental disorders, genetic syndromes, and mild intellectual disabilities were more prevalent among non-affective psychosis patients.

Most participants in both groups (over 60%) originated from urban areas. Regarding family structure, 56.58% of affective psychosis patients came from stable, biparental families, compared to 46.32% of non-affective patients. Re-institutionalization was observed in 5.26% of non-affective patients, while no cases were reported in the affective psychosis group.

Academic performance varied, with 35.53% of affective psychosis patients achieving good grades and 10.53% dropping out, compared to 26.32% and 22.11% in non-affective psychosis, respectively.

**Figure 3:**
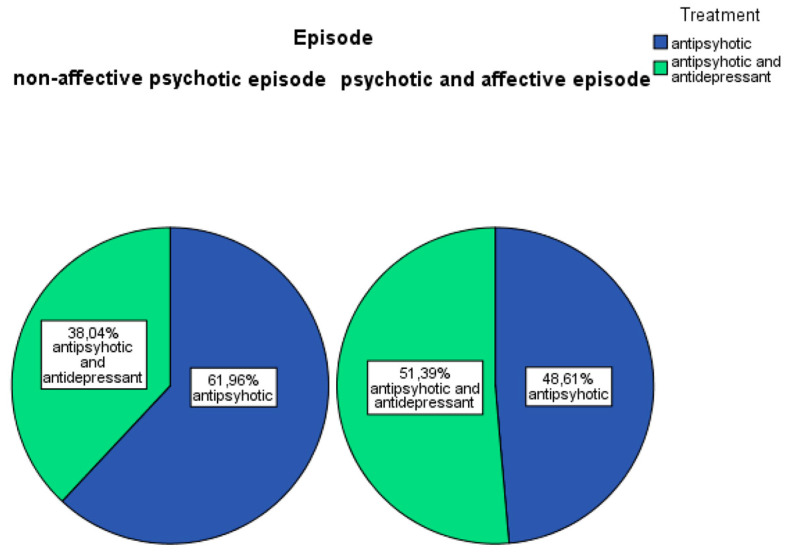
Treatment.

Treatment patterns differed: 61.96% of non-affective psychosis patients received antipsychotics alone, and 38.04% received combined antipsychotic and antidepressant therapy. Among affective psychosis patients, 48.61% received antipsychotics alone, and 51.39% received combination therapy (antipsychotic + antidepressant) (Figure 3).

Benzodiazepines were commonly used for the management of agitation and aggression, as well as for addressing sleep disturbances, with both indications accounting for over 60% of their overall use.

**Figure 4:**
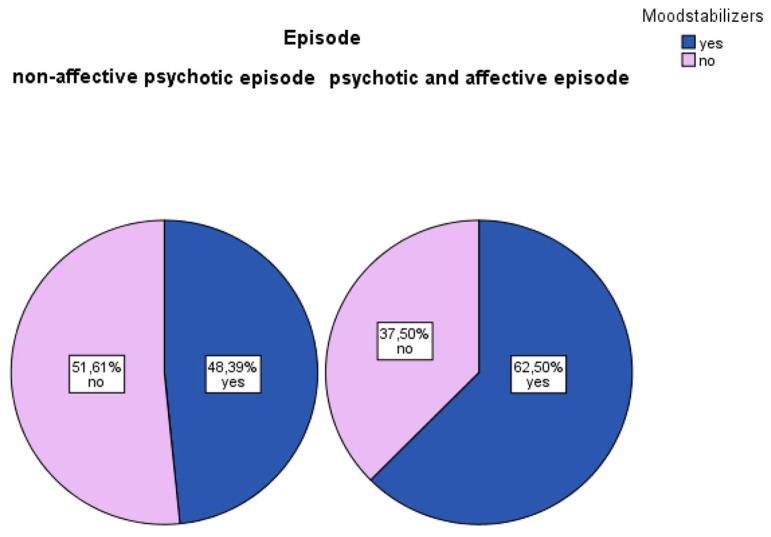
The need for mood stabilizer.

The use of mood stabilizers was selected for 62.5% of the group suffering from affective psychosis, with some variability observed among its subcategories: 48.72% for individuals exhibiting depressive symptoms and 77.42% for those displaying manic symptoms. Meanwhile, 48.39% of the adolescents in the non-affective psychotic group were given a mood stabilizer (Figure 4).

Table 1 presents the sociodemographic, clinical, and treatment characteristics of patients with non-affective and affective psychosis.

### 3.2. Statistical Analysis

#### 3.2.1. Gender and Type of Psychotic Episode

Chi-square analysis revealed a significant association between gender and type of psychotic episode (χ^2^ = 4.395, *p* = 0.036).

#### 3.2.2. Psychiatric Family History

No significant association with episode type (χ^2^ = 1.284, *p* = 0.257).

#### 3.2.3. Somatic Family History

No significant association with episode type (χ^2^ = 0.094, *p* = 0.759).

#### 3.2.4. Place of Origin

No significant association with episode type (χ^2^ = 0.079, *p* = 0.778).

#### 3.2.5. Family Structure

No significant association with episode type (χ^2^ = 5.779, *p* = 0.328).

#### 3.2.6. School Performance

No significant association with episode type (χ^2^ = 4.671, *p* = 0.457).

#### 3.2.7. Initial Treatment Type

No significant association with episode type (χ^2^ = 2.921, *p* = 0.087).

#### 3.2.8. Benzodiazepine Use

No significant association with episode type (χ^2^ = 0.196, *p* = 0.658).

#### 3.2.9. Mood Stabilizer Use

Mood stabilizer use was more frequent in AP, particularly in manic episodes (observed trend), but this did not reach statistical significance (χ^2^ = 3.260, *p* = 0.071).

#### 3.2.10. Age Groups

No significant association with episode type (χ^2^ = 1.441, *p* = 0.487).

### 3.3. Multivariate Analysis

We performed an exploratory multiple logistic regression model in order to check if the significant results found at univariate analysis were still significant when adjusted for other covariates (Table 2). We included all the previous predictors, excepting family structure. Treatment-related variables such as receiving antipsychotic + antidepressive, benzodiazepines, or mood stabilizers were not included. They are highly correlated with the diagnostic and would risk circular interpretation of the results. These two variables have multiple categories that cannot be structured ordinally. We wanted to respect the rule of 10 events per variable established by Peduzzi et al. [12] so that our model would yield reliable results. Although the overall model reached statistical significance, the low McFadden R^2^ indicates limited explanatory power. Male gender was less likely to develop a psychotic disorder with affective symptoms compared to female gender. Bootstrapped coefficients were calculated for internal validation; the 95% confidence interval for the gender predictor did not include 1, confirming the results remained consistent.

## 4. Discussion

The results indicate several descriptive findings that warrant further exploration in future research. Our study found a significant association between gender and type of psychosis, with women more frequently experiencing affective psychosis and men more frequently experiencing non-affective psychosis. These patterns are consistent with previous literature. However, they should be interpreted cautiously, as our retrospective, single-center design does not allow causal inferences or determination of underlying biological mechanisms [13,14,15].

A 2022 systematic review and meta-analysis by Carter et al. synthesized data from 35 studies examining sex and gender differences in early psychosis, reporting that men exhibit more severe negative symptoms, whereas women display more pronounced depressive symptoms and higher levels of functioning [16]. Similarly, Choi et al. (2009) and the EPPIC cohort (Cotton et al., 2009) reported gender differences in symptom patterns and functional outcomes [17,18,19,20]. These findings provide context for our observations without implying mechanistic explanations.

Regarding family and personal history, our data suggest a trend toward higher rates of alcohol dependency and anxiety–depressive traits in families of adolescents with affective psychosis compared to the non-affective group. No significant associations were found between personal pathological events and type of episode, and although literature suggests potential neurodevelopmental links for non-affective psychosis, our findings cannot confirm this due to limited data and the retrospective design. Observed patterns, such as increased institutional care for NAP patients, highlight areas for future research, including attachment and psychosocial development [8,21,22].

Academic performance may reflect premorbid functioning. In our sample, affective psychosis was more often associated with good academic performance, whereas non-affective psychosis showed higher rates of absenteeism or dropout, though these differences were not statistically significant. Existing literature links cognitive deficits to non-affective psychosis, but evidence regarding affective psychosis remains limited [21,23]. Our study cannot determine whether poor academic performance represents premorbid functioning or prodromal features.

Treatment patterns in our cohort were generally consistent with published recommendations. Atypical antipsychotics were commonly used in both groups. Mood stabilizers were more frequently prescribed in affective psychosis, reflecting a trend toward addressing affective symptoms, although this association did not reach statistical significance. Short-term use of benzodiazepines did not differ by episode type. Observations from Consoli et al., 2009, and WHO mhGAP, 2023, support the preferential use of mood stabilizers in adolescents with affective psychotic features [4,11,19,20,24,25,26,27]

The exploratory multivariate analysis confirmed the significant role of gender as a predictor of affective psychosis. No other variables were significantly associated with episode type. Mood stabilizer use was observed more frequently in the affective group, but this finding should be interpreted as a descriptive trend rather than a predictive association.

Overall, our study does not provide a definitive clinical profile for affective or non-affective psychosis, but highlights descriptive differences related to gender and treatment patterns. These findings are exploratory and suggest directions for future research, including investigation of risk factors, functional outcomes, and tailored intervention strategies. The single-center and retrospective design should be considered when interpreting these results.

## 5. Limitations

Our study has several limitations that should be considered when interpreting the findings. First, the retrospective design relies on medical records and caregiver interviews, which may be incomplete or biased, particularly for patients from foster care or institutional settings. Second, there were no standardized methods for quantifying certain variables, such as academic performance or the recollection of family psychiatric history, which may limit comparability and generalizability. Third, diagnostic criteria can vary over time and across practitioners, potentially introducing classification bias. While we did not observe any cases of patients transitioning from AP to NAP before the age of 18, the retrospective design and limited follow-up do not allow us to exclude such changes completely. Finally, our study is single-center and exploratory, so associations observed in univariate and multivariate analyses should be interpreted cautiously, as other unmeasured confounding factors may influence the outcomes. These limitations highlight the need for prospective, multi-center studies with standardized assessments to confirm and expand upon our findings.

## 6. Conclusions

In this single-center, retrospective study of adolescents with psychotic episodes, we observed descriptive differences between individuals with affective psychosis (AP) and non-affective psychosis (NAP). Female gender was significantly associated with AP, while male gender was more frequently observed in NAP. Mood stabilizer use was observed more often in AP, reflecting real-world clinical treatment patterns; however, this finding did not reach statistical significance and should be interpreted as a descriptive trend.

Other variables, including academic performance, family structure, and most neurodevelopmental or environmental factors, showed descriptive patterns but did not achieve statistical significance. Multivariate logistic regression confirmed the significance of gender, while mood stabilizer use was retained in the description as a trend rather than a predictive variable.

Overall, these findings are exploratory and descriptive. They highlight patterns that may inform future research, guide clinical awareness, and assist in early identification and management of adolescents with psychotic features. However, causal inferences cannot be drawn due to the retrospective and single-center design. Future multi-center, prospective studies with standardized assessments are needed to clarify the clinical, developmental, and functional profiles of AP and NAP in adolescence.

## Figures and Tables

**Table 1:** Characteristics of Patients with Non-Affective and Affective Psychosis.

Variable	Non-Affective Psychosis	Affective Psychosis
Number of patients	95 (55.5%)	76 (44.5%)
Male (%)	62.77%	37.23%
Female (%)	46.75%	53.25%
Mean age (years)	15.39 ± 1.617	15.39 ± 1.617
Psychiatric family history	48 (50.53%)	46 (59.21%)
No personal medical history	22 (23.16%)	28 (36.84%)
Urban origin	60 (61.05%)	49 (63.16%)
Stable biparental family	44 (46.32%)	43 (56.58%)
Re-institutionalization	5 (5.26%)	0 (0%)
Good academic performance	25 (26.32%)	27 (35.53%)
School dropout	21 (22.11%)	8 (10.53%)
Antipsychotics only	59 (61.96%)	37 (48.61%)
Combination therapy (antipsychotic + antidepressant)	36 (38.04%)	39 (51.39%)
Mood stabilizer use	46 (48.39%)	48 (62.5%)

**Table 2:** Multiple logistic regression model including all significant predictors from univariate analysis.

Variable	Odds (OR)	*p*-Value	CI Lower	CI Upper
Whole model		0.029		
Age	1.216	0.060	0.992	1.491
Gender (male)	0.504	0.038	0.264	0.963
Neuropsychiatric history (No)	0.664	0.210	0.350	1.259
Urban origin	1.199	0.587	0.623	2.305
School result (enrollment)	2.376	0.065	0.947	5.963

## Data Availability

The data presented in this study are available on request from the corresponding author due to privacy and ethical restriction.
